# Parasitic Infections: A Role for C-Type Lectins Receptors

**DOI:** 10.1155/2013/456352

**Published:** 2013-01-27

**Authors:** Alicia Vázquez-Mendoza, Julio César Carrero, Miriam Rodriguez-Sosa

**Affiliations:** ^1^Unidad de Biomedicina, Facultad de Estudios Superiores-Iztacala, Universidad Nacional Autónoma de México, 54090 Tlalnepantla, MEX, Mexico; ^2^Departamento de Inmunología, Instituto de Investigaciones Biomédicas, Universidad Nacional Autónoma de México, 70228 Ciudad de México, DF, Mexico

## Abstract

Antigen-presenting cells (APCs) sense the microenvironment through several types of receptors that recognize pathogen-associated molecular patterns. In particular, C-type lectins receptors (CLRs), which are expressed by distinct subsets of dendritic cells (DCs) and macrophages (MØs), recognize and internalize specific carbohydrate antigens in a Ca^2+^-dependent manner. The targeting of these receptors is becoming an efficient strategy for parasite recognition. However, relatively little is known about how CLRs are involved in both pathogen recognition and the internalization of parasites. The role of CLRs in parasite infections is an area of considerable interest because this research will impact our understanding of the initiation of innate immune responses, which influences the outcome of specific immune responses. This paper attempts to summarize our understanding of the effects of parasites' interactions with CLRs.

## 1. Introduction

Lectins are a diverse group of mono- and multivalent proteins and glycoproteins of nonimmune origin that have selective affinity for a carbohydrate or a group of carbohydrates [1]. These proteins are widely distributed in plants, animals, and microorganisms. In animals, lectins have been identified in a great number of cells. Lectins are either embedded in intracellular or cell surface membranes or are present in a soluble form in the plasma. Inside cells, lectins are also found in the cytosol and in the nucleus. Animal lectins play a crucial role in both physiological and pathological processes. Specific interactions between lectins and complex carbohydrates (glycoproteins, glycolipids, polysaccharides, or proteoglycans) are involved in numerous basic phenomena, such as embryonic development, intracellular trafficking, cell-cell and cell-matrix recognition, cell homing, endocytosis, phagocytosis, inflammation, and the metastatic spread of cancer cells (Table 1) [2].

## 2. Structural Characteristics of C-Type Lectin Receptors

**Figure 1:**
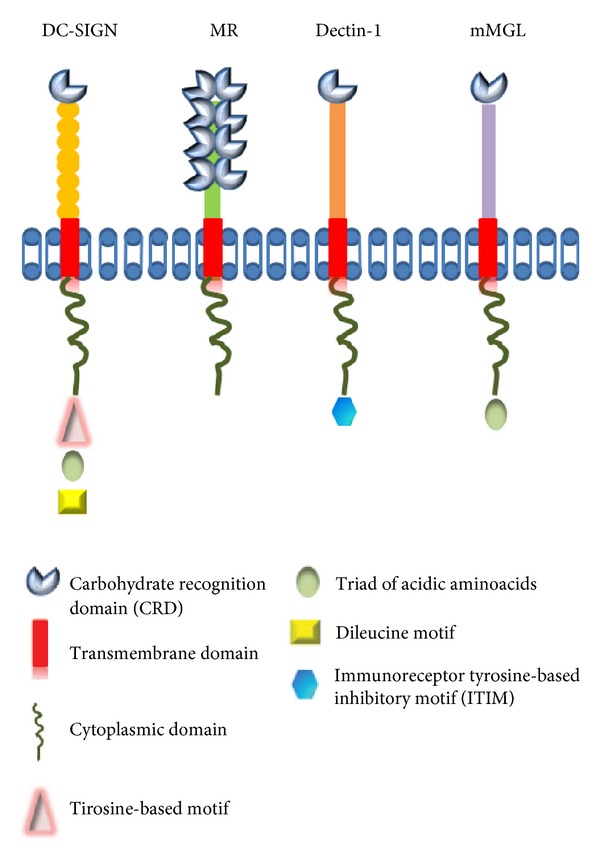
Structure of members of the C-type lectin (DC-SIGN, MR, Dectin1, and MGL). These receptors contain one or more carbohydrate-recognition domain (CRD), transmembrane domain, and cytoplasmic domain may contains tyrosine-based motif, triad of acidic amino acids, dileucine motif or immunoreceptor tyrosine-based inhibitory motif.

The CLRs constitute a superfamily of more than 1,000 proteins classified into 17 groups based on their phylogeny and domain organization. Most CLRs possess one or more carbohydrate recognition domains (CRDs) or C-type lectin-like domains (CTLDs). The CTLD is a conserved structural motif containing as two protein loops stabilized by two disulfide bridges at the base of each loop. The second loop is more flexible than the first and generally contains the ligand binding site. Most CLRs are membrane-associated receptors that are involved in antigen capture and presentation [25, 26]. Endocytosis mediated by CLRs is guided by their intracellular internalization motifs, whereas some CLRs contain ITIM (immunoreceptor tyrosine-based inhibitory motif)-or ITAM (immunoreceptor tyrosine-based activation motif)-like motifs in their cytoplasmic domains, illustrating the potential immune-suppression or immune-activation functions of these receptors (Figure 1) [27]. 

Based on the primary structure of their CRDs, their folding patterns, and their cation requirements, animal lectins can be classified into several families, including C-, F-, P-, and I-type lectins, galectin, pentraxin, and others (Table 1) [18]. However, the most important molecules from the CLR family include macrophage galactose-type C-type lectin (MGL), dendritic cell-specific intercellular adhesion molecule-3-grabbing nonintegrin (DC-SIGN), the mannose receptor (MR), DEC205, and Dectin-1 (Figure 1). 

## 3. Role of C-Type Lectin Receptors in the Immune Response

The initial recognition of an invading pathogen by antigen-presenting cells APCs, such as macrophages (MØs) or dendritic cells (DCs), is crucial in determining the type of effector T cell that subsequently mediates an immune response [1, 2]. APCs are equipped with highly specialized receptors, including an array of pattern recognition receptors (PRRs), such as C-type receptors (CLRs) and Toll-like receptors (TLRs). These receptors play an important role in the activation/maturation of APCs upon binding with conserved pathogen structures known as pathogen-associated molecular patterns (PAMPs). In contrast to TLRs, CLRs recognize and internalize specific carbohydrate antigens expressed by pathogens and host tissues in Ca^2+^-dependent manner [18, 25–27].

Protein-carbohydrate interactions have important roles in two distinct aspects of the immune response. These interactions are involved both in pathogen recognition and in the cellular interactions that lead to pathogen neutralization [28]. Lectin receptors play an important role in the innate immune response by recognizing and binding specific carbohydrate moieties (usually a nonreducing terminal monosaccharide or oligosaccharide) on the surface of potential pathogens through CRDs [1, 2]. CRDs, in combination with other domains, can recognize carbohydrate moieties and induce agglutination, immobilization, complement-mediated opsonization and lysis [25]. 

In this review, we focus on integral membrane C-type lectins and their participation in the recognition of glycosylated parasite antigens. Despite the presence of a highly conserved domain, C-type lectins are functionally diverse and have been implicated in various processes, including cell adhesion, tissue integration and remodeling, platelet activation, complement activation, pathogen recognition, endocytosis, and phagocytosis [3, 29, 30].

The importance of C-type lectins is highlighted by the fact that several pathogens and tumor antigens take advantage of these receptors to escape intracellular degradation and to suppress the generation of an efficient immune response [31]. Several studies have demonstrated that some C-type lectins may function as adhesion, signaling, or antigen-uptake receptors [32–35], and these results are consistent with the fact that some CLRs are present on MØs and DCs, which play a role in the initial step of capturing the antigens containing carbohydrates [36].

Several CLRs have been shown to contribute to the loading of endocytosed antigens on MHC class I and class II, thereby facilitating effective antigen-specific CD4^+^ and CD8^+^ T-cell responses [37, 38]. There are evidence that some CLRs (like DC-SING, DC205, and Dectin-1) are able to trigger distinct signaling pathways that modulate cell functions through the expression of specific molecules and cytokines, in most cases promote the antigen presentation and determining the polarizations of T cells [4, 39]. However, most evidence about CLR trigger signaling pathways have emerged using virus, bacterial pathogens, fungus, or peptides. There are not evidence about the interaction of parasites with CLRs and activation of a signaling pathway.

The signaling through MGL is emerging recently, using DCs, has been demonstrated that MGL engagement to anti-MGL antibody or MUC1_9Tn_ triggered the phosphorylation of ERK1,2 and the activation of NF-kB signal promoting DC activation and increase in antigen-specific CD8^+^ T-cell activation; however, the effects of this activation are strongly dependent on the type of stimulus added to the cells [5]. 

Moreover, several studies suggest that CLRs may also modulate immune reactions through cross-talk with other receptors, especially TLRs. These results indicate that the outcome of an immune response is determined by the balance between triggering the two receptors families [5, 40]. Many transmembrane C-type lectins belonging to groups II, V, and VI are expressed primarily by myeloid cells. Although many are “orphan” receptors, others have been shown to promote the phagocytosis of nonopsonized microbes and to induce cytokine production in MØs and DCs, leukocytes that play critical roles in innate immunity and in the subsequent modulation of adaptive immune responses [41]. These properties make the C-type lectin family an optimal tool for APCs to target parasites. 

## 4. C-Type Lectins in Parasitic Infection (Table 2)

A number of glycan moieties have been identified in most parasites that potentially bind various CLRs, which act as sensors of the innate immune system.

### 4.1. Protozoa

#### 4.1.1. *Leishmania*

The trypanosomatid flagellates of the genus *Leishmania* cause diverse diseases with varying clinical symptoms and underlying pathologies. These diseases include visceral leishmaniasis (Kala-azar), mucocutaneous leishmaniasis, cutaneous leishmaniasis, and post-Kala-azar dermal leishmaniasis (PKDL) [42].

These diseases cause significant morbidity and mortality in the 98 countries or territories, where they are endemic [43]. *Leishmania* have two developmental stages: the promastigote, which is an extracellular flagellated form that is transmitted by insect vectors, and the amastigote, which is an intracellular multiplicative form that multiplies within the phagocytes of the vertebrate host, a process that involves different ligand-receptor systems [44]. The repetitive structure and glycan modifications associated with many *Leishmania* cell surface molecules suggest that these parasites may interact with CLRs, for example, MR and DC-SIGN [6, 45].

*Mannose Receptor (MR)*. MR is a C-type lectin. It is a transmembrane glycoprotein (175 kDa) with eight C-type-lectin-like domains (or carbohydrate-recognition domains, CRDs) that is expressed on the surface of several cell types, such as MØs, DCs, and some epithelial cells. MR mediates the binding and internalization of mannosylated glycoproteins and participates in the endocytosis of different pathogens bearing mannose residues on their surfaces [6, 46, 47]. 

Previous studies both *in vivo* and *in vitro* have demonstrated the involvement of MR during the recognition and internalization of promastigotes of different *Leishmania* species (*donovani, amazonensis*). Mouse peritoneal MØs infected with *L. donovani* exhibited a decrease in MR activity, with a loss of 50% of original binding activity after 4 days of infection. A possible explanation for this decrease in the expression of MR is the direct correlation with the number of amastigotes within MØs and the recovery of MR activity after the elimination of parasites from MØs after treatment with methotrexate/mL conjugated with bovine serum albumin modified with mannose (Man-BSA) for 3 h [48]. Competition assays with different MR ligands (Man-BSA or D-mannose) revealed an important decrease in the activity of MR, with a loss between 50% to 80% in phagocytic capacity, demonstrating the participation of MR during parasite recognition and the upregulation of MR expression during the initial steps of the infection [6, 35, 48, 49]. 

A recent study showed that bone marrow-derived macrophages (BMDMs) infected with *L. major* metacyclic promastigotes exhibit TNF-*α* and IL-12 production levels similar to those in MR-wild-type (MR-WT) mice and MR-knockout (MR-KO) mice. The clinical course of *L. major* and *L. donovani* infections was slightly different with respect to the area covered by lesions between the MR-WT and MR-KO mice at week 7. However, the levels of ulcer healing and the resolution of the lesions were equivalent. Moreover, assays measuring the activation of MAPKs (ERK1/2, p38, and JNK) revealed that MR is not necessary for the inhibition of ERK and p38 activation. In addition, immunohistochemical analysis of cutaneous lesions from MR-KO and MR-WT mice revealed no differences in lesion architecture or cell components. Together, these data suggest that MR is not essential for host resistance against *Leishmania* infections and that either redundant MØ receptors compensate for the lack of MR or MR does not play a role in parasite attachment [45].

*Dendritic Cell-Specific Intercellular Adhesion Molecule-3-Grabbing Nonintegrin (DC-SIGN)*. Also known as CD209, DC-SIGN is a type II transmembrane CLR that is expressed on DCs and involved in cell-cell interactions through its capacity to bind ICAM-3 and ICAM-2 [50, 61]. This receptor is used by protozoan parasites of the genus *Leishmania*. Previous studies have investigated possible *Leishmania*/DC-SIGN interactions through the use of fluorescence-labeled parasites in combination with blocking agents such as anti-DC-SIGN antibodies and soluble mannan. These studies showed that DC-SIGN is a receptor for the promastigotes and amastigotes of both the visceral (*L. infantum*) and cutaneous (*L. pifanoi*) forms but not for *Leishmania major *metacyclic promastigotes, suggesting that DC-SIGN is a broad *Leishmania* receptor that exhibits variable affinity for distinct infective forms and species of the parasite [50, 51]. There is no doubt that these findings are important; however, it remains to be determined whether this recognition influences the immune response to* Leishmaniasis.*

#### 4.1.2. *Trypanosoma cruzi*

The protozoan parasite *Trypanosoma cruzi *(*T. cruzi*), the etiological agent of human Chagas disease, is endemic in Latin America, where 18–20 million of people are infected [62]. Infection leads to an acute phase that may last between 2 and 4 months and is characterized by high numbers of parasites in the bloodstream and tissues. The control of parasite replication leads to chronic, often life-long disease. Most individuals in the chronic phase have a silent, asymptomatic clinical form of Chagas' disease and are classified as indeterminate patients [63]. However, approximately 30% of chronically infected individuals develop a severe clinical form in which digestive and/or cardiac alterations often lead to death [64–67]. 

During the process of parasite internalization, the interaction between receptors expressed in the host cell and the parasite is important because these receptors are responsible for recognizing the major antigens of *T. cruzi*. This parasite expresses mucin-like glycoproteins (TcMUCs) on its membrane. These proteins are highly glycosylated glycoconjugates (approximately 60% of their weight is carbohydrates) and are threonine-rich, serine- and proline-rich polyanionic molecules that are anchored to the plasma membrane through glycosylphosphatidylinositol [64, 68, 69]. 

Furthermore,* T. cruzi *contains a major lysosomal cysteine proteinase called cruzipain (Cz), one of the immunodominant antigens of *T. cruzi*. Cz is a glycoprotein of approximately 52–58 kDa and has both high mannose and complex type-N-linked glycans in the C-terminal domain. It is expressed in all stages of the parasite and is highly immunogenic in humans. Moreover, it has been shown that Cz induces the alternative activation of MØs *in vitro* and upregulates arginase activity. This activation profile was shown to be associated with the functional ability of these cells to promote the intracellular growth of *T. cruzi *[46].

*Mannose Receptor (MR).* Enzyme binding assays using HRP (horseradish peroxidase) as the mannosylated ligand, which were used to characterize the cardiomyocyte mannose receptor (CM-MR) and its involvement in *T. cruzi* invasion, demonstrated that after the infection of cardiomyocytes (CM) with *T. cruzi*, a considerable reduction in HRP binding was noticed. Binding was almost completely restored by treating the infected cultures with the trypanocidal drug nifurtimox [52]. These results showed that CM-MR participated in the adhesion and uptake of *T. cruzi* by CM.

Another study found that *T. cruzi*-infected MØs pre-incubated with mannose-bovine serum albumin (Man-BSA, MR specific ligand) exhibited high levels of urea, increased intracellular amastigote growth, the downregulation of JNK and p44/p42 phosphorylation, and an increase in p38 MAPK phosphorylation relative to control cells. In addition, MØs incubated with Cz or Man-BSA exhibited enhanced MR recycling. However, *T. cruzi*-infected peritoneal MØs incubated with an MR-blocking antibody showed reductions in arginase activity and intracellular parasite growth. Moreover, the level of MR on peritoneal cells from *T. cruzi*-infected BALB/c mice at 13 and 15 days after-infection has been evaluated, and flow cytometry analysis revealed an increase in F4/80^+^ MR^+^ cells as the infection progressed. Together, these results showed that the interaction with MR on MØs may be a mechanism by with *T. cruzi* evades the innate immune response both *in vitro* and *in vivo* [46].

#### 4.1.3. *Trypanosoma brucei*

The protozoan parasite *Trypanosoma brucei (T. brucei)* is the causative agent of the human and animal African trypanosomiasis, which is frequently fatal if not treated. This parasite has a digenetic life cycle, replicating in the alimentary canal of its vector, the tsetse fly, and in the bloodstream of mammals. In the mammalian host, the bloodstream form of *T. brucei *lives and divides extracellularly in the blood, lymph, and interstitial fluids [70, 71]. The bloodstream form of *T. brucei *is rich in galactose-containing glycoproteins, most notably the abundant variant surface glycoprotein (VSG), which protects the parasite from the complement pathway and undergoes antigenic variation to evade specific immune responses [72]. 

*Macrophage Galactose Type C-Lectin (MGL)*. MGL is a member of the type II family of C-type lectins and has an approximate molecular mass of 42 kDa. MGL is expressed on immature human and mouse DCs and MØs in the skin and lymph nodes [27, 73]. Mice contain two functional copies of the MGL gene, mMGL1 and mMGL2 [74], which are both expressed by dermal DCs and MØs [53, 75], whereas in humans, only one MGL gene is found [76]. mMGL1 and mMGL2 have different carbohydrate specificities: mMGL1 is specific for Lewis X (Le^x^) and Lewis^A^ structures, whereas mMGL2, similar to hMGL, recognizes *α*/*β*-GalNAc structures and galactose, including O-linked Tn-antigen, TF-antigen, and core 2 structure [54, 77]. In the skin, MGL is a marker for CD1a^+^ dermal DCs, a cell type with enhanced ability to stimulate naive T cells relative to other dermal APC subsets. 

Raes et al. report that mMGL1 and mMGL2 are induced in peritoneal MØs during *in vivo* infection with *T. brucei*, correlating with a switch from a type I cytokine environment in the early stage of infection to a type II cytokine environment in the late and chronic phases. In addition, it has been demonstrated that the incubation of thioglycolate-elicited peritoneal MØs with IL-4 o IL-13 moderately induced mMGL1 expression and strongly induced mMGL2 expression, but IFN-*γ* did not [53]. The results presented in this paper suggest that the mMGL1 and mMGL2 receptors are novel markers for type II cytokine-dependent alternatively activated macrophages (aaMØ) both *in vitro* and in the chronic phase of infection with *T. brucei. *These findings are important, but the possible interaction between antigens of *T. brucei* and mMGL remains to be defined, as does the role of mMGL in the immune response.

### 4.2. Nematodes

#### 4.2.1. *Trichuris muris*

Several gastrointestinal nematodes have been reported to express ligands for MR on their surface. *Trichuris muris *(*T. muris*) is a natural mouse model of the gastrointestinal nematode parasite *Trichuris trichiura *(*T. trichiura*), one of the most prevalent human helminth infections. Studies of the role of cells in immune responses to *T. muris *and the mechanisms of immune expulsion of these worms from mice have demonstrated that B cells and antibodies are required for resistance to thisparasite. The evasion of the immune response by *T. muris* causes chronic infection, which has the ability to manipulate the host immune system. *T. muris* excretory/secretory (E/S) products from a heterogeneous solution of worm proteins contain substances that have been shown to bear mannose and N-acetylglucosamine residues; therefore, these substances are potential ligands for C-type lectin receptors such as MR [78]. 

Deschoolmeester et al. showed *in vitro* that MR-KO-derived bone-marrow-derived MØs (BMDMs) expressed similar levels of several cytokines when exposed to *T. muris* E/S. The only difference observed was a reduction in the production of IL-6 by alternatively activated BMDMs in the absence of MR, and the infection of MR-KO mice revealed the expulsion of *T. muris* with the same kinetics as observed for WT animals and a similar cytokine response in the draining mesenteric lymph nodes. Moreover, there were no differences in MØ recruitment, the ability of MØs to become alternatively activated, goblet cell hyperplasia, or gross crypt pathology during infection. In summary, MR binds to components of *T. muris*, but it is not required for the development of an immune response leading to the expulsion of *T. muris* [47].

### 4.3. Helminths: Trematodes

#### 4.3.1. *Schistosoma mansoni*

Parasitic helminths express various carbohydrates containing glycoproteins on their surface and release glycan-rich E/S products that can potentially bind to various CLRs [59]. The parasite helminth *Schistosoma mansoni (S. mansoni) *is the causative agent of the chronic disease schistosomiasis, which is the second most prevalent human parasitic disease, affecting *∼*300 million people worldwide, particularly in tropical countries [55, 79]. Immunologically, *S. mansoni *infection is dominated by two distinct Th phases: an initial Th1 (IFN-*γ*) response, which switches to a stronger Th2 (IL-10, IL-5, and IL-13) response [58]. One of the most striking features of schistosomiasis is that the worms are experts in modulating and evading the host immune response, enabling their survival, migration, and development in different host tissues. Schistosomal glycoconjugates (glycoproteins and glycolipids) have shown to play important roles in host-parasite interactions. These glycoconjugates are often developmentally regulated antigens that are expressed during different life cycle stages. Some studies have indicated that Lewis^X^ antigens Gal*β*1,4(Fuc*α*1–3)GlcNAc have important roles in host-schistosome interactions. Lewis^X^ (Le^x^) antigens have been found in glycoconjugates from all life cycle stages, including the membrane-bound glycoproteins of adult schistosomes and secreted egg and gut glycoproteins [7].

*Macrophage Galactose Type C-Lectin (MGL)*. Human MGL has an exclusive specificity for terminal GalNAc residues, such as those found in the glycoproteins of the helminth parasite *S. mansoni*, in filoviruses, and in tumor-associated antigens [80]. 

Binding assays revealed that MGL recognizes both terminal *β*-GalNAc residues of LDN [GalNAc*β*1–4GlcNAc-R] and LDNF [GalNAc*β*1–4(Fuc*α*1–3)GlcNAc-R] glycans present in SEA of *S. mansoni*. The specific interaction between MGL and SEA glycoproteins containing LDN and LDNF demonstrates that MGL functions as a pattern recognition receptor for *S. mansoni *[54].

In another study using binding assays and blocking antibodies reported that SEA of *S. mansoni* is internalized by human DCs through MGL. Moreover, the confocal laser scanning microscopy reveals colocalization of SEA with MHC-II in the lysosomal compartments suggests that Ag processing and presentation can occur. Certainly these findings are important, however remains to be answered if this recognition leads to antigen presentation and modulation of the immune response to *S. mansoni* [55].

*DC-SIGN*. It has been demonstrated that the blockade with monoclonal antibodies against the carbohydrate antigens Le^x^ and LDNF inhibit the binding of DC-SIGN to soluble egg antigens (SEAs). The glycoproteins several SEAs from different schistosome species (*S. mansoni, S. haematobium, and S. japonicum*) contain ligands for DC-SIGN. It has also been demonstrated that a specific mutation in the carbohydrate-recognition domain (CDR) of DC-SIGN abrogates binding to either SEAs or Le^x^ [56].

Structural characterization of the glycolipids and the study of cellular binding revealed that DC-SIGN binds to the carbohydrate moieties of glycosphingolipids with Le^x^ and Le^y^ structure [Fuc*α*1-2Gal*β*14(Fuc*α*1–3)GlcNAc] moieties. DC-SIGN recognizes not only the self-glycan ligand Le^x^ within cercarial glycolipids, but also glycolipids carrying pseudo-Le^y^, a nonself-structure that to date has been found within S*chistosome cercarial (S. cercarial) *glycolipids and ES products [7]. These results show that DC-SIGN recognizes Le^x^ and Le^y^ antigens present in the SEAs and glycolipids of *S. cercarial*. Thus, DCs likely interact with S*chistosomes* early during infection through this lectin. However, more studies are needed to determine whether the recognition of glycosylated antigens through DC-SIGN is involved in resistance or susceptibility to* S. mansoni* infection *in vivo.*

*L-SIGN.* Liver/lymph node-specific ICAM-3-grabbing nonintegrin (LSIGN/CD209L/DC-SIGN-R) is a human homolog of DC-SIGN. L-SIGN shares 77% amino acid sequence identity with DC-SIGN and is expressed on liver sinusoidal endothelial cells (LSECs), which function as antigen-presenting cells in the liver [81]. 

L-SIGN, a highly related homolog of DC-SIGN, can bind both schistosome egg antigens (SEAs) and glycosphingolipids and can mediate the internalization of SEAs. However, binding assays showed that L-SIGN recognizes a glycoprotein fraction different from that recognized by DC-SIGN. It has been demonstrated that L-SIGN does not bind to neoglycoconjugates carrying Le^x^ but does recognize other fucosylated glycans, that is, Le^(a,b and y)^. Other studies have demonstrated that the glycosylation of schistosome antigens plays an important role in immunological process during schistosome infection [8, 57]. Those studies confirmed that L-SIGN recognizes both oligomannosidic N-glycans and multiply fucosylated carbohydrate motifs within SEAs. In addition, these studies demonstrated that L-SIGN can recognize a broad but specific glycan profile.

*SIGNR1*. Also called CD-209b, is one of the eight mouse homologs of human DC-SIGN and is expressed on particular MØ subsets in the marginal zone of the spleen and the medulla of the lymph nodes and on the peritoneal MØs. SIGNR1 recognizes glycans from different pathogens and has been shown to bind Lewis^x/y^ and Lewis^a/b^-containing carbohydrates [82, 83].

 An *in vitro* studyusing cells transfected with SIGNR1 showed that glycans from both SEAs and schistosome worm antigens were bound by SIGNR1 in a dose-dependent manner, demonstrating the ability of SIGNR1 to recognize and bind to two different stages of the parasite. However, the *in vivo* infection of SIGR1-deficient BALB/c mice (SIGNR-KO) with 25 cercariae of *Schistosoma* revealed that SIGNR1 has no role in primary or secondary pulmonary granuloma induced by schistosome eggs. SIGNR-KO mice exhibited unaltered worm fecundity, and the fecal eggs and the size and eosinophil content of the granulomas surrounding eggs in the liver were comparable, as were the levels of hepatic fibrosis. Moreover, no differences in the cytokine the production by spleen cells were observed. In conclusion, although SIGNR1 can recognize *S. mansoni* antigens *in vitro*, this receptor does not have a functional role *in vivo* during infection [9].

*Dectin-2*. Dectin-2 is a member of the C-type lectin family and has single complementarity-determining region (CRD). This protein expressed mainly in MØs and DCs. Dectin-2 recognizes *α*-mannans and transduces the signal through an association with the ITAM-containing Fc receptor *γ* chain [84, 85].

*In vitro* restimulation assays using spleen and MLN cells with SEA (20 *μ*g/mL) have demonstrated that SEA associates with Dectin-2 and Fc receptor *γ*-chain (FcR*γ*) receptors. Moreover, SEA-mediated IL-1*β* production was significantly inhibited when BMDCs were pretreated with Dectn-2-specific antibodies or when Dectin-2-deficient BMDCs were used. In contrast, TNF-*α* production was not impaired. Thus, different components within SEAs mediate different immune reactions. These observations suggest that SEA triggers the Dectin-2 receptor, which couples with FcR*γ* chain, to activate the Syk-kinase signaling pathway, which controls IL-1*β* release in an ROS- and potassium efflux-dependent manner, the Nlrp3 inflammasome activation, and IL-1*β* release. However, even though these findings are important, it is necessary to determine whether this receptor plays a key role during infection *in vivo* [58]. 

*Mannose Receptor (MR).* It has been demonstrated that infective larvae of the parasitic helminth *S. mansoni* contain a large number of glycosylated components specific for MR. MR ligands are particularly rich in excretory/secretory (E/S) material released during the transformation of cercariae into schistosomula, a process that is critical for infection of the host. E/S material from carboxyfluorescein diacetate succinimidyl ester (CFDA-SE)-labeled cercariae showed enhanced binding by Chinese hamster ovary cells (CHO) lines transduced to express MR and by an MØ cell line that overexpresses MR (J774E) relative to the level of binding by WT CHO cells. Conversely, uptake was significantly lower by bone marrow-derived macrophages (BMDM) from MR-KO mice, although these cells were more active as judged by the enhanced proinflammatory cytokine production and CD40 expression. After natural percutaneous infection of MR-KO mice with CFDA-SE-labeled parasites, there were fewer cells in the skin and draining lymph nodes that were CFDA-SE(+) relative to the numbers in WT mice, indicating that there was reduced uptake and presentation of larval parasite antigens. However, the antigen-specific proliferation of skin-draining lymph node cells was significantly enhanced, and these cells secreted markedly elevated levels of IFN-*γ* but decreased levels of IL-4. These results demonstrated that MR on mononuclear phagocytic cells plays a significant role in internalizing E/S material released by the invasive stages of the parasite, which in turn modulates the production of proinflammatory cytokines. In the absence of MR, antigen-specific CD4^+^ cells are Th1 biased, suggesting that the ligation of MR by glycosylated E/S material released by schistosome larvae modulates the production of IFN-*γ* by CD4^+^ cells [59].

### 4.4. Helminths: Cestodes 

#### 4.4.1. *Taenia crassiceps*

*Taenia crassiceps (T. crassiceps)* is a tapeworm that is found in wild and domestic animals but does not cause clinical disease in nonimmunocompromised humans. This parasite has been used as an experimental model for cysticercosis [86]. Previous studies demonstrated that soluble antigens from *T. crassiceps* are highly glycosylated and are responsible for Th2 polarization *in vivo* [87, 88]. One study found that the excretory/secretory products of the cestode *T. crassiceps* (TcESs) do not induce the maturation of human DCs, as demonstrated by the lack of increase in the expression levels of CD83, HLA-DR, CD80, and CD86. TcESs enhanced the production of IL-10, positively modulated the expression of mMGL and negatively modulated the expression of DC-SIGN, although the source of these antigens is not a human parasite. These results showed that TcESs induce a tolerogenic-like phenotype in human DCs and modulate the expression of PRRs involved in key functions of DCs such as mMGL and DC-SIGN. This modulation is a possible mechanism used by *T. crassiceps* to modify the phenotype and hence the functions of human DCs, directing the balance toward immune suppression and allowing the survival of this parasite [60].

## 5. Conclusion

All studies described above demonstrate that CLRs are essential to the recognition of different carbohydrates present on surface or in the excretory/secretory products of different parasites. This recognition can promote the uptake, internalization and processing of parasite antigens that can influence the immune response. However, little is known about the role of CLRs in the immune response to parasitic infections. Future studies are needed to understand the immune mechanisms underlying the interaction of parasite antigens with CLRs.

## Figures and Tables

**Table 1:** Summary of structural and functional properties of the lectin family receptors.

Group	Molecules structure	Family members	Ligands	Expression	Function	Reference
		MR	Mannose, fucose, and N-acetylglucosamine	MoPh, retina DCs, LCs, Fbls, and kidney	Pathogen recognition, antigen presentation, clearance of endogenous cytopathic molecules, and regulation of circulating hormones	[3–14]
		DC-SIGN	Mannose, ICAM-3	Mesangial cells and CMs, MØ, and DCs	Pathogen recognition, antigen presentation, cell migration, and DC-T-cell interactions	
C-type	Type-I Type-II (Tm)	SIGNR-1	Zymosan, mannans, and dextran	iDCs spleen MZ, lymph node, and pMØ	Clearance of blood borne antigens	
		Dentin1	*β*-glucans	DCs, neutrophils, and splenic T cells	Antifungal host defense, induction of TNF-*α*, and regulation of T-cell proliferation	
		Dectin2	*α*-mannans	MØ, DCs	Impairment of UV-induced tolerance	
		mMGL1	Gal	MØ, DCs	Internalization and antigen presentation, bind to CD45 to inhibit T cells	
		mMGL2	Sructure Le^x^	MØ, DCs	Anti-inflammatory response	
		L-SIGN	Structure Le^(a,b,y)^	Liver sinusoidal endothelial cells	Antigen receptor	

P-type	Type-I (Tm)	CD-MPR CI-MPR	Man-P-GlcNAc Man-6-P	Lysosomal hydrolases	Transport Man-6-P containing acid hydrolases from the Golgi to endosomal/lysosomal compartments	[15–17]

F-type		AAA MsaFBP32	Fucose	Liver and kidney	Modulation of cell functions	[18–22]

I-type	Type-I (Tm)	Siglec-1 Siglec-2 Siglec-4 Siglec-15	Sialic acids with N- and O-linked glycosylations	Myeloid and lymphoid cells	Regulation of cell signaling from leucocytes	[23, 24]
		Siglec-3 10 members humans (3, 5, 6, 7, 8, 9, 10, 11, 14, 16) Rodents Siglec-3, E, F, G, H			Endocytic receptors	

Abbreviations: Tm: transmenbrane; MØ: macrophages; pMØ: peritoneal macrophages; Dcs: dendritic cells; iDCs: immature dendritic cells; MoPh: mononuclear phagocytes; Fbls: fibroblasts; LCs: langerhans cells; CMs: cardiomyocytes; Le^x^: Lewis x, a, b, and y structures; Gal: galactose; MR: mannose receptor; DC-SIGN: dendritic cell-specific ICAM-3-grabbing nonintegrin; SIGNR-1: SIGN-related 1; homologe DC-SIGN; mMGL: macrophage galactose type c-lectin; L-SIGN: liver/lymph node-specific ICAM-3 grabbing nonintegrin; CD-MPR: cation-dependent mannose 6-phosphate receptor; CI-MPR: cation-independent mannose 6-phosphate receptor; Man-6-P: mannose 6-phosphate; Man-P-GlcNAc: mannose 6-phisphate N-acetylglucosamine ester; AAA: Anguilla anguilla agglutinin; MsaFBP32: F-lectin present in striped bass (Morone saxatilis).

**Table 2:** C-type lectins in parasitic infection.

Parasite	Receptor	Model	*In vivo/in vitro *	Role	Reference
Protozoo					
*L. donovani *	MR	BALB/c mice	*in vivo/ in vitro *	Uptake of mannose containing glycoconjugates	[48]
	MR	Swiss albino mice	*in vitro *	Binding promastigotes	[36]
	MR	hmDMØ	*in vitro *	Attachment and ingestion promastigotes	[49]
*L. amazonensis *	MR	Skin Fbls	*in vitro *	Uptake of mannosylated ligands	[6]
*L. major *	MR	BMDMs MR-KO mice	*in vivo *	Recognizes mannose residues on the surface *Leishmania*, but it's not essential for host defense	[45]
*L. pifanoi *	DC-SIGN	MDDCs cell line K562	*in vitro *	Binding and internalization of amastigotes	[50]
*L. pifanoi* *L. infantum *	DC-SIGN	IMDDCs cell lineK562	*in vitro *	Receptor for promastigotes and amastigote infective stages from both visceral and cutaneous leishmaniasis	[51]
*T. cruzi * *Tulahuen strain *	MR	BALB/c mice Cell line J774 (MØ)	*in vivo/in vitro *	Bind to Cz, increasing MR recycling which leads to arginase activity	[46]
Y and DM strains	MR	CM and MØ	*in vitro *	Adhesion and uptake of parasites	[52]
*T. brucei *	MGL	C57BL/6 mice BALB/c mice	*in vivo *	Marker of aaMØ	[53]
Nematodes					
*T. muris *	MR	C57BL/6 MR-KO mice	*in vivo/in vitro *	Recognized components E/S of parasites	[47]
Trematodes					
* S. mansoni *	MGL	Cell lines SW948, SKBR3, ZR75-1 CHO, BLM, FM3.29 FM6, SK23mel	*in vitro *	Recognized LDN and LDNF glycans	[54]
	MGL	Human DCs	*in vitro *	Internalization of glycolipids of SEA	[55]
	DC-SIGN	Human DCs	*in vitro *	Adhesion to glycolipids of SEA	[7]
	DC-SIGN	Human DCs	*in vitro *	Recognize glycans of SEA	[56]
	L-SIGN	Cell line K562	*in vitro *	Binds to structures Le^a,b,y^ of SEA	[8]
	L-SING	Cell line K562	*in vitro *	Binds and internalization of SEA	[57]
	SIGNR1	BALB/c WT or SIGNR1-KO	*in vivo/in vitro *	Recognize antigens of AWA and SEA	[9]
	Dectin-2	C57BL/6	*in vivo/in vitro *	Binds SEA component	[58]
	MR	C57BL/6 WT or MR-KO	*in vivo/in vitro *	Internalization E/S material by schistosome larvae	[59]
Cestodes *T. crassiceps *	MGL DC-SIGN	Human DCs	*in vitro *	TcES positively modulated the expression of MGL but negatively modulated DC-SIGN	[60]

Abbreviations: MR: mannose receptor; DC-SIGN: dendritic cell-specific ICAM-3 grabbing nonintegrin; SIGNR-1: SIGN-related 1; homologe DC-SIGN; mMGL: macrophage galactose type c-lectin; L-SIGN: liver/lymph node-specific ICAM-3 grabbing nonintegrin; Fbls: fibroblasts; BMDMs: bone marrow-derived macrophages; MDDCs: monocyte-derived dendritic cells; IMDDCs: immature monocyte-derived DCs; MØ: macrophages; CM: cardiomyocyte; Cz: cruzipaina; E/S: excretory/secretory; LDN: [GalNAc*β*1–4GlcNAc-R]; LDNF: [GalNAc*β*1–4(Fuc*α*1–3)GluNAc-R]; SEA: soluble egg antigens; Le: structures of Lewis; AWA: adult worm antigen; TcES: *Taenia crassiceps* excreted-secreted antigens.
